# Perturbing cortical networks: *in vivo* electrophysiological consequences of pan-neuronal chemogenetic manipulations using deschloroclozapine

**DOI:** 10.3389/fnins.2024.1396978

**Published:** 2024-04-25

**Authors:** Péter Kovács, Lauren N. Beloate, Nanyin Zhang

**Affiliations:** ^1^Department of Biomedical Engineering, Pennsylvania State University, University Park, PA, United States; ^2^Huck Institutes of the Life Sciences, Pennsylvania State University, University Park, PA, United States; ^3^Center for Neural Engineering, Pennsylvania State University, University Park, PA, United States; ^4^Center for Neurotechnology in Mental Health Research, Pennsylvania State University, University Park, PA, United States

**Keywords:** neuronal oscillations, DREADD = designer receptor exclusively activated by designer drugs, chemogenetics, deschloroclozapine, GAD67, *in vivo* electrophysiology, DCZ, rat – brain

## Abstract

**Introduction:**

Chemogenetic techniques, specifically the use of Designer Receptors Exclusively Activated by Designer Drugs (DREADDs), have become invaluable tools in neuroscience research. Yet, the understanding of how Gq- and Gicoupled DREADDs alter local field potential (LFP) oscillations in vivo remains incomplete.

**Methods:**

This study investigates the in vivo electrophysiological effects of DREADD actuation by deschloroclozapine, on spontaneous firing rate and LFP oscillations recorded from the anterior cingulate cortex in lightly anesthetized male rats.

**Results:**

Unexpectedly, in response to the administration of deschloroclozapine, we observed inhibitory effects with pan-neuronal hM3D(Gq) stimulation, and excitatory effects with pan-neuronal hM4D(Gi) stimulation in a significant portion of neurons. These results emphasize the need to account for indirect perturbation effects at the local neuronal network level in vivo, particularly when not all neurons express the chemogenetic receptors uniformly. In the current study, for instance, the majority of cells that were transduced with both hM3D(Gq) and hM4D(Gi) were GABAergic. Moreover, we found that panneuronal cortical chemogenetic modulation can profoundly alter oscillatory neuronal activity, presenting a potential research tool or therapeutic strategy in several neuropsychiatric models and diseases.

**Discussion:**

These findings help to optimize the use of chemogenetic techniques in neuroscience research and open new possibilities for novel therapeutic strategies.

## Introduction

Chemogenetics, an innovative gene therapy tool, involves the expression of genetically engineered receptors on specific cells within the central nervous system (CNS) through viral transduction (Roth, 2016; Aldrin-Kirk and Björklund, 2019; Poth et al., 2021). This method allows precise control over specific brain areas or neural networks through systemic administration of otherwise inert small molecule drugs, termed “actuators”, which in turn, can inhibit or excite the targeted cells with high spatial and temporal precision. Based on these characteristics, chemogenetic methods offer a unique research tool for neuroscientists (Smith et al., 2021), and hold the potential to revolutionize CNS medicine, presenting an advanced alternative to current pharmacotherapy and deep brain stimulation (DBS) methods in various neurological and psychiatric disorders (Urban and Roth, 2015; Poth et al., 2021).

There is a lack of data and understanding of how exactly chemogenetic applications alter local network electrophysiological functionality and output in different brain areas, *in vivo*. While hM4D(Gi) and hM3D(Gq) DREADDs are commonly used to silence and activate neurons, respectively (Roth, 2016), this concept is generally based on molecular (Armbruster et al., 2007) or cellular-level evidence, and typically validated by *in vitro* electrophysiological means (Nentwig et al., 2022). While *in vitro* electrophysiology is invaluable, it remains important to confirm these intended effects of hM4D(Gi) and hM3D(Gq) DREADDs with *in vivo* recordings from a functioning neuronal network. In fact, there are several reports based on *in vivo* recordings showing counterintuitive electrophysiological effects, such as neuronal excitation caused by hM4D(Gi) stimulation (Chang et al., 2015; Deffains et al., 2021) and firing rate inhibition caused by hM3D(Gq) stimulation (Vazey and Aston-Jones, 2014; Rogers et al., 2021). These, so-called ‘perturbation’ effects are hypothesized to be consequences of imperfect DREADD expression *in vivo*, meaning that not all neurons are expressing the exogenous receptors in the network, and therefore might modify the overall electrophysiological output of the area and consequently alter the overall behavioral effects of the chemogenetic intervention (Smith et al., 2016). Finally, even less is understood on how exactly *in vivo* chemogenetic manipulations can modify the local oscillatory neuronal networks.

To address the above detailed gaps in our knowledge, the goal of the current experiment was to investigate the *in vivo* electrophysiological effects of deschloroclozapine (DCZ)-actuation of the two most commonly used DREADD receptors, hM4D(Gi) and hM3D(Gq), on cortical spontaneous firing rate and LFP oscillations. Most importantly, we aimed to validate the general concept, that hM4D(Gi) stimulation predominantly silences, while hM3D(Gq) stimulation mostly activates neurons in a given cortical brain area. To do so, we utilized multichannel *in vivo* extracellular electrophysiological recordings (spontaneous firing activity and LFP oscillations) from the anterior cingulate cortex (ACC), in lightly anesthetized rats. The ACC is an ideal target area to study cortical network dynamics because it is an important center regulating whole brain networks, including its oscillatory function that was shown to affect learning, memory, and cognition (Womelsdorf et al., 2010; Taub et al., 2018). Furthermore, ACC has been demonstrated to shape resting state networks, thus acting as one of the most important cortical hub areas (Tu et al., 2021a,b; Ma et al., 2022). Since our focus of investigation was the *in vivo* network dynamics at the local cortical network level, which can be positioned above the molecular/cellular level-, but below the systemic/behavioral organizational level in biological systems, a low dose isoflurane-induced sedation was our preferred recording environment. This enabled us to record high quality spiking activity, from a stable and spontaneously active local network (Vazey and Aston-Jones, 2014; Deffains et al., 2021), while eliminating the confounds of behavioral- or movement-related signals and a low signal-to-noise ratio. We applied the viral constructs by neurosurgical means, with the utilization of a pan-neuronal promoter (i.e., the human synapsin promoter, hSyn). Additionally, we aimed to investigate the short-term *in vivo* electrophysiological pharmacodynamics of systemically applied DCZ as an advanced actuator of choice, to provide further support as an alternative option to CNO in DREADD-related experiments.

## Materials and methods

### Animals

The data in the present study were collected from 9 adult male Long-Evans rats (307–574 g). The rats were procured from Charles River Laboratory (Wilmington, MA) and were acclimated in their home cages for a period of 7–10 days before undergoing the stereotaxic surgical procedure. The rats were housed in Plexiglas cages and had access to standard lab chow and water *ad lib.*, under a 12-h light and 12-h dark cycle, with the temperature set to 22–24 degrees Celsius. All experiments conducted in this study received approval from the Pennsylvania State University Institutional Animal Care and Use Committee.

### Chemogenetic viral transduction

After the initial acclimation, aseptic stereotaxic surgery was performed to introduce the viruses into the brain. Rats were initially anesthetized with isoflurane, followed by intramuscular injections of ketamine (Midwest Veterinary Supply, Lakeville, MN; 40 mg/kg) and xylazine (12 mg/kg). Subcutaneous (s.c.) injections of dexamethasone (0.5 mg/kg) and enrofloxacin (2.5 mg/kg) were given to prevent tissue inflammation and bacterial infections. Buprenorphine (1.0 mg/kg) was injected subcutaneously to induce post-surgery analgesia. After exposing the skull, small holes were drilled towards the area of interest and the following injections were administered into the ACC:

- hM3D(Gq) group, *n* = 3: pAAV-hSyn-hM3D(Gq)-mCherry (AAV8 serotype);
- hM4D(Gi) group, *n* = 3: pAAV-hSyn-hM4D(Gi)-mCherry (AAV8 serotype);
- Control group, *n* = 3: Sham surgery with phosphate-buffered saline (PBS) injection.

Viral expression was under the control of the hSyn promoter, effectively limiting the DREADD receptor expression solely to neurons. This method is also referred to as ‘pan-neuronal’ targeting, since all neuronal subtypes (e.g., inhibitory interneurons and pyramidal cells) are targeted with the hSyn promoter (Wang et al., 2023). The viral volumes and titers and the stereotaxic coordinates for viral injections were as follows: 3 × 1 μL virus or control solution was injected towards ACC: +1.5 mm A/P, +0.5 mm M/L and − 3.8, −2.8 and − 1.8 mm D/V (Paxinos and Watson, 2014). Viral solutions were injected undiluted at ≥2 × 10^12^ vg/mL [hM3D(Gq)] and ≥ 7 × 10^12^ vg/mL [hM4D(Gi)] viral titers, using an automated Stoelting™ Quintessential Stereotaxic Injector (Stoelting Co., Wood Dale, IL) with the utilization of a 5 μL capacity Hamilton syringe (Neuros Syringe, Model 75 RN, 33 gauge, Point Style 4, Hamilton Co. Reno, NV). Viral vectors of pAAV-pAAV-hSyn-hM3D(Gq)-mCherry and pAAV-hSyn-hM4D(Gi)-mCherry were purchased from Addgene (plasmids #50474 and #50475, respectively; Addgene Co., Watertown, MA). The craniotomy was filled with a surgical silicone adhesive (Kwik-Cast, World Precision Instruments, Sarasota, FL) and the skin was closed above the wound. Rats remained in homecages for at least 7 weeks to recover and to allow for proper viral transduction prior to the subsequent electrophysiological recording session.

### Electrophysiological recordings and DCZ administration

To induce an initial anesthesia, 3–4% isoflurane was applied followed by a single low dose injection of xylazine (5 mg/kg, i.p., AnaSed Injection, Akorn, Lake Forest, IL). During this deeper anesthetic state, the animal’s head was fixed into the stereotaxic frame, the previous head incision was re-opened, and the surgical silicone adhesive was removed from the craniotomy that was drilled during the viral injection. To record neuronal action potentials and local LFPs, a 16-channel silicon electrode array probe (type: A1x16-5 mm-150-177, NeuroNexus Co., Ann Arbor, MI) was slowly dropped into the ACC (AP: +1.5, ML: +0.5, DV: −3.8). We are confident that the electrode placement overlapped considerably with the virus expression, as the craniotomy from the viral injection surgery was still present and aligned with the stereotaxic coordinates at the time of electrophysiological recordings. The 16 recording channels were located 150 μm distance from each other on the probe’s surface, covering a 2.25 μm-long dorsoventral recording area (DV: approx. -3,8–1.55 mm), fully spanning the previous 3 viral injection sites (Supplementary Figure S1). The reference wire of the probe was connected to the probe ground and together they were inserted carefully into a small craniotomy drilled at the contralateral hemisphere. The reference wires were gently touching the brain surface/dura, without causing any damage to the brain or bleeding, and the instrumental ground wire was connected to the stereotaxic frame. After the initial surgical preparations had been completed, a much lower, sedation-level dose of isoflurane (0.3–0.5%) was set and a light anesthesia was maintained during the subsequent ~1 h recording session.

After recording a stable 20+ min baseline spontaneous firing activity from the ACC, we administered a s.c. injection of 0.3 mg/kg DCZ. We used a water soluble DCZ dihydrochloride salt (Hello Bio Co., #HB9126), which was easily and perfectly dissolved in sterile saline. We used a freshly made DCZ solution for each stimulation.

### Data processing

Each recorded electrode channel was separately analyzed (single neuronal firing activity and LFP analyses) from every animal, due to our observation that the 150 μm distance from each neighboring recording channels were sufficiently large to record from completely separate single neuronal spiking networks (for a demonstration recording sample please see Supplementary Figure S2).

The electrophysiological signal was sampled at 20 kHz and amplified using a NeuroNexus recording system, with a SmartBox data acquisition device (NeuroNexus, Ann Arbor, MI). The acquired 16-channel raw electrophysiology data was converted to Spike2 software format (*.smr files) and analyzed with the help of the Spike2 program (Spike2, version 7.00, Cambridge Electronic Design Co., Cambridge, UK). A notch filter was applied at 60 Hz for each channel. The following 7 Infinite Impulse Response (IIR) second order band-pass Butterworth digital filtering passing limits (Zhang et al., 2020) were applied to each of the 16 recorded channels to separate 6 LFP band-specific channels and 1 Spiking channel from each raw data channel, for further analysis:

- Spiking channel to analyze single neuronal firing activity – Low Pass: 500 Hz, High Pass: 2 kHz;
- Gamma band – low pass: 40 Hz, High Pass: 100 Hz;
- Beta band – low pass: 12 Hz, High Pass: 40 Hz;
- Alpha band – low pass: 8 Hz, High Pass: 12 Hz;
- Theta band – low pass: 4 Hz, High Pass: 8 Hz;
- Delta band – low pass: 1 Hz, High Pass: 4 Hz;
- Unfiltered LFP channel – only notch filtering.

Single neuronal spikes were separated from the continuous digitalized spiking channel, using a ± 25 μV spike amplitude discriminator and further classified using the built-in spike separator function in the Spike2 program, based on action potential amplitude and waveform shape differences. The separated single neuronal data channels were then individually analyzed. First, 1 s-based frequency histograms were built and then changes in firing rates (in Hz, 1/s) after DCZ activation were evaluated. We used 3-min single neuronal firing rate average values as the basis of the statistical analyses, except for determining the type of neuronal response (inhibition-, excitation- etc.) where we used the 1 s-based firing rates. To determine the type of neuronal responses to stimulus, we applied two-sample unequal variance t-test to each post-stimulus 3-min window and compared them to the 1–4 min pre-stimulus baseline firing rates. The modality of single neuronal effects was determined based on significant firing rate changes compared to the pre-stimulus baseline activity. The following neuronal response modalities were specified: excitation (E), inhibition (I), excitation-inhibition (EI), excitation-inhibition-excitation (EIE), inhibition-excitation (IE), inhibition-excitation-inhibition (IEI), excluded or no effect (*N*). The main exclusion criterion was defined as a significant difference between the pre-stimulus 1–4 vs. 4–7 min baseline activity (unstable baseline, which served as a recording quality check), but the 1–4 vs. 7–10, and the 1–4 vs. 10–13 min differences were also taken into consideration in more complicated cases.

### Experimental design and statistical analysis

Single neuronal effects were analyzed statistically in a collided (all neurons added together) and a separated (neurons with similar effects added together) manner, applying one-way ANOVA, Tukey HSD post-hoc tests and dependent samples t-tests (Statistica version 6.1, StatSoft Inc. Tulsa, OK).

LFP band-specific power data (in μVolt^2^) was extracted from the separated band-specific channels from Spike2 (see details above), using pre- and post-stimulus 12-min windows (Pre 1–13, Post 1–13, 13–25, 25–37 min) and was further analyzed using Microsoft 365 Excel (Microsoft Co., Redmond WA) and Statistica programs, using mainly factorial ANOVA and LSD post-hoc tests. Additionally, a normalized LFP band specific power data was calculated from dividing the post-stimulus band specific power data by the pre-stimulus power. ANOVA and LSD post-hoc tests were also carried out on the normalized data set.

Spiking-correlated power data (in μVolt^2^) was similarly extracted from the separated band-specific channels with Spike2, using the same method as described above (LFP band-specific power data extraction), with the difference that only the ±200 ms peri-spike intervals were extracted, not the whole recording session. This approach led to the creation of a distinct dataset exclusively focused on LFP activity changes synchronized with the neural spiking activity. Data analysis methods were the same as in the case of the LFP band-specific power data (see above).

### Tissue processing and histology

Following electrophysiological recordings, animals received an overdose of 2:1 ketamine/xylazine. They were then transcardially perfused with 60–120 mL saline [0.9% NaCl (Sigma-Aldrich) in ddH2O] and ~ 240 mL 4% paraformaldehyde [Electron Microscopy Sciences; in 0.1 m phosphate buffer (PB)]. Brains were removed and postfixed for 1 h in the same fixative at room temperature prior to being stored in a sucrose solution [Thermo Fisher Scientific; 20% in 0.1 m PB containing 0.01% sodium azide (Sigma-Aldrich)] at 4°C for at least 24 h.

Brains were then sectioned coronally (35 μm) on a freezing stage microtome (Leica Biosystems SM2010 R) into four parallel series. Three of the series were stored in cryoprotectant solution [30% sucrose in 0.1 m PB containing 30% ethylene glycol (Thermo Fisher Scientific) and 0.01% sodium azide] at 4°C for future processing, and one series was immediately mounted onto Superfrost plus glass slides (Fisher Laboratories) and allowed to dry prior to Fluoroshield with DAPI (Sigma-Aldrich) application and coverslipping.

In a second series containing the ACC, free-floating sections were incubated and washed at room temperature under gentle agitation, in 0.1 M PBS, for at least 4 h prior to additional processing and between all incubations. Sections were then exposed to 1% H_2_O_2_ (10 min; in PBS) and incubated in blocking solution [2.5 h; PBS containing 0.1% bovine serum albumin (Thermo Fisher Scientific) and 0.4% Triton X-100 (Sigma-Aldrich)]. Sections were then incubated in mouse anti-GAD67 17 h; (EMD Millipore; 1:2,000) and goat anti-mouse conjugated to Dyelight 488 (2.5 h; Invitrogen; 1:100), followed by rabbit anti-RFP (17 h; Rockland; 1:20,000) and goat anti-rabbit conjugated to Alexa 555 (30 min; Invitrogen; 1:100). Sections were mounted on Superfrost plus glass slides (Fisher Laboratories) and coverslipped with Fluoroshield with DAPI (Sigma-Aldrich).

All sections including the ACC were visually examined for mCherry fluorescence or mCherry and GAD67 using an inverted fluorescence phase-contrast microscope (Keyence BZ-9000 E). In the first series, locations of viral injections were mapped and images were taken using a Texas-R/mCherry filter for viral expression visualization and a DAPI filter for cell nuclei visualization using the BZ-II Viewer software at 4x, 10x and 20x magnification. In the second series, sections including the ACC were imaged using a Texas-R/mCherry filter for visualization of viral expression, a DAPI filter for cell nuclei visualization, and a GFP filter for GAD67 visualization using the BZ-II Viewer software at 20x magnification. BZ-II Analyzer software was used to adjust image brightness, contrast and fluorescent blur, overlay channels and add scales. Three sections from each animal in hM3D(Gq) and hM4D(Gi) groups were chosen, and a 200 μm x 200 μm area within the ACC was used to manually quantify number of DAPI+ cells, number of mCherry+ cells, number of GAD67+ cells, number of mCherry+/GAD67+ cells, percentage of GAD67+ cells that co-express mCherry and percentage of mCherry+ cells that co-express GAD67.

## Results

### Effects on spontaneous firing rate, spiking-oscillation coupling and up-down LFP dynamics

#### hM3D(Gq) effects

**Figure 1:**
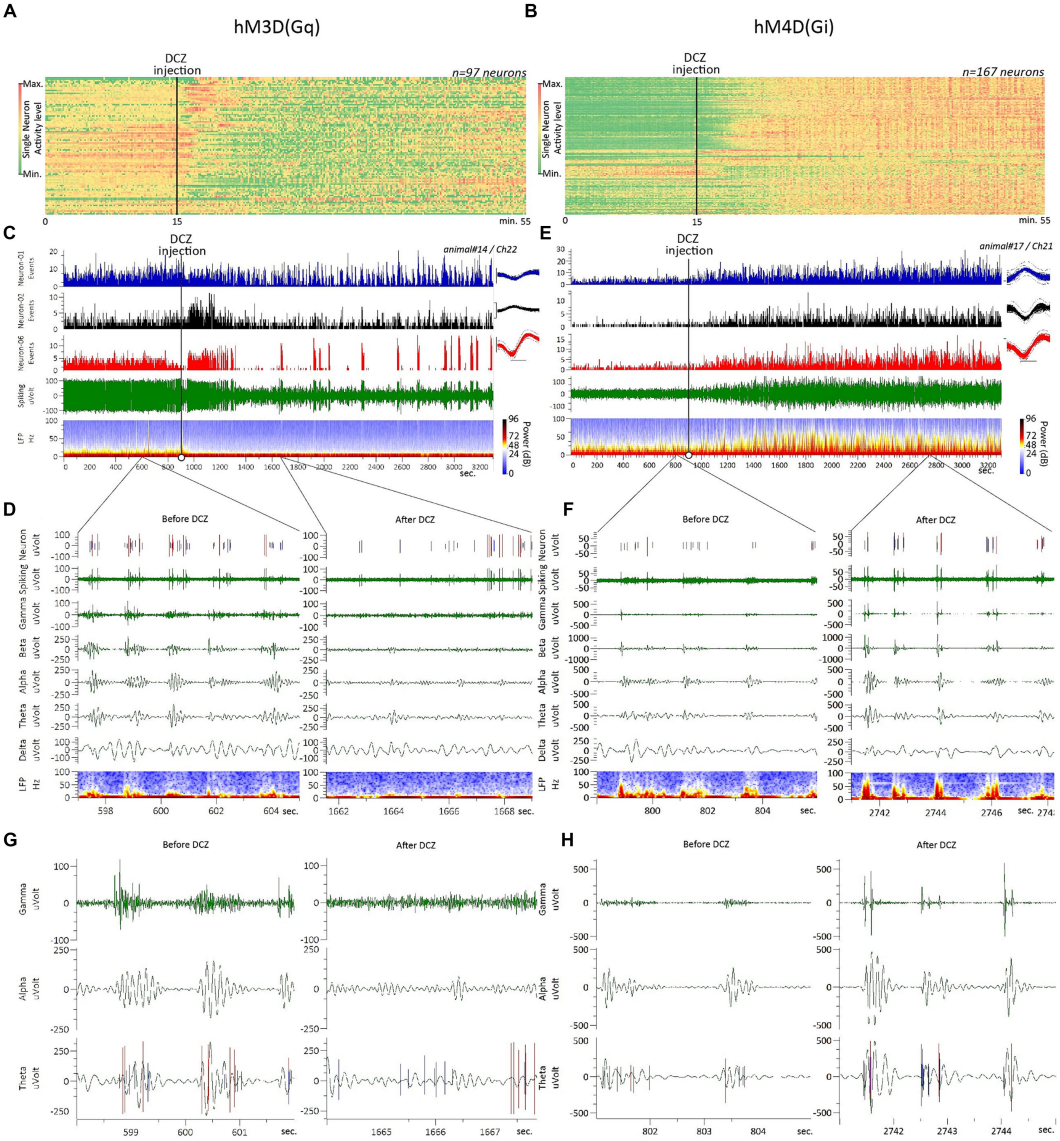
Raw electrophysiology data and typical neuronal responses to hM3D(Gq) and hM4D(Gi) DREADD receptor stimulation. **(A,B)** Raster diagram displays the raw firing rates of all neurons recorded from the ACC of rats transfected with hM3D(Gq) and M4D, respectively. **(C,E)** Typical recording raw data sample showing the firing rate, LFP and band-specific oscillatory activity changes in response to DCZ stimulation, recorded from rats transfected with hM3D(Gq) and M4D, respectively; spike waveform averages on the inserts in **C,E** are represented by the overdrawn waveform of 100 spikes, scale bar dimensions: x-axis 0.4 msec., y-axis 50 μVolt. **(D,F)** Close-up view on the raw data samples (represented in **C,E**), focusing on the second-scale changes before (left side) and after (right side) DCZ stimulation. **(G,H)** A larger magnitude, sub-seconds-scale close-up view, with the spiking channel superimposed on the Theta oscillations channel, allowing a more detailed observation of spiking-oscillations correlations, before (left side) and after (right side) DCZ stimulation. Please note that the scaling of the band specific LFP channels on **D,G** vs. **F,H** are scaled differently to allow clearer observation of the different directional changes in LFP power, in response to DCZ stimulation.

We recorded and analyzed ACC neurons (*n* = 97) from hM3D(Gq)-transfected rats (*n* = 3) in response to DCZ administration. Neuronal inhibition was the most common response observed, sometimes initiated by transient excitation followed by a long-term inhibitory change in firing activity (Figures 1A,C). This typical biphasic response is demonstrated in Figure 1C, in a raw data sample, where, out of the three recorded neurons, two respond with a fast (<1 min post-DCZ injection) initial excitation that lasts for a few minutes, followed by a longer-term neuronal inhibition. On the raster diagram (Figure 1A), where the raw firing rates of all recorded neurons from hM3D(Gq)-transfected rats can be seen, a clear inhibitory response is evident, starting a few minutes after the DCZ injection and lasting more or less until the end of the 55-min recording session.

Upon closer examination of the typical spiking and oscillatory consequences of a DCZ injection on hM3D(Gq)-transfected rats (Figures 1D,G), we observed a phenomenon known as ‘up-down’ cortical dynamics that is evident in firing activity before the DCZ administration (Jercog et al., 2017). Cortical neurons only fire within the “up” states during this baseline period (Figures 1D,G, left panels). However, after DCZ activation, both the up-down dynamics and the oscillatory power itself diminish, and neurons simultaneously start firing outside of the “up” states (Figures 1D,G, right panels).

**Figure 2:**
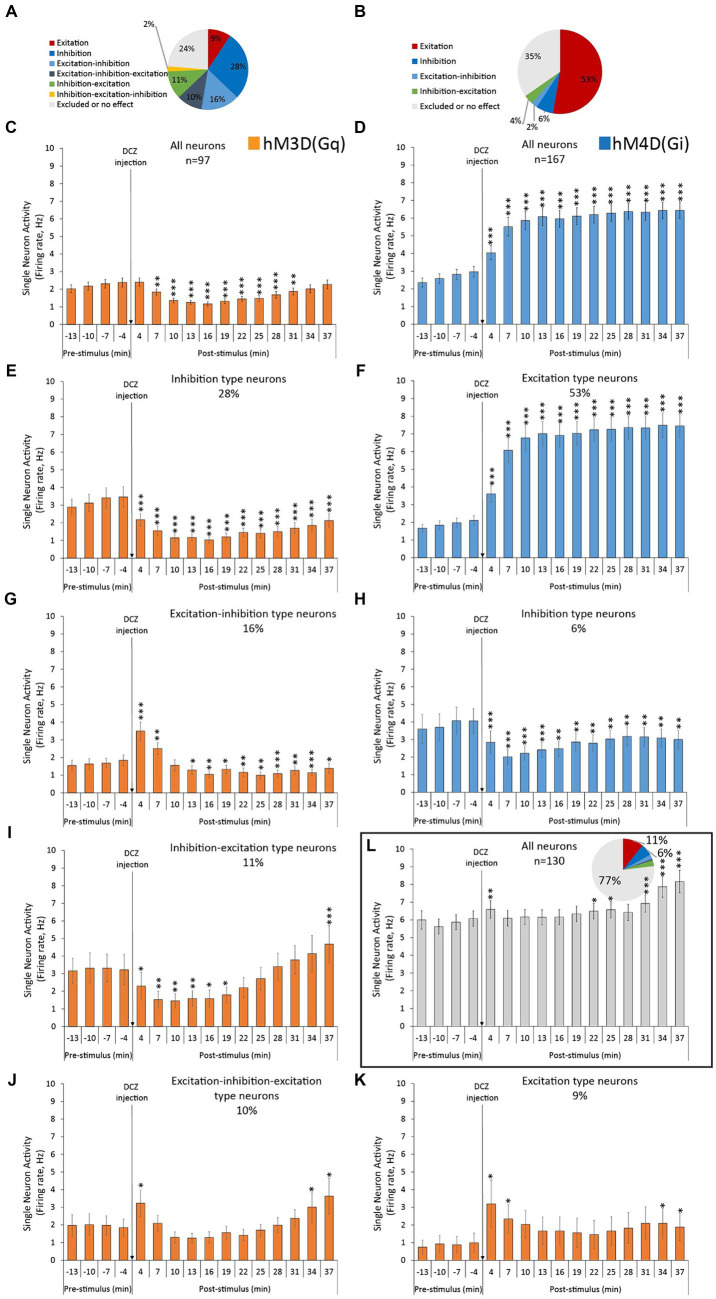
Categorized electrophysiological effects of hM3D(Gq) and hM4D(Gi) DREADD receptor stimulation on the spontaneous single neuron firing activity, recorded from the ACC. **(A,B)** Percentage distribution of the recorded neurons, based on their response modality. **(C,D)** Averaged neuronal responses to DCZ stimulation of all recorded neurons. **(E,G,I–K)** Averaged neuronal responses to DCZ stimulation of the different subclasses of neurons, based on their response modality, recorded from hM3D(Gq)-transfected animals. **(F,H)** Averaged neuronal responses to DCZ stimulation of the different subclasses of neurons, based on their response modality, recorded from hM4D(Gi)-transfected animals. **(L)** Averaged neuronal responses to DCZ stimulation of all control neurons, and their percentage distribution based on their response modalities (insert), recorded from Sham-operated animals, with no DREADD receptors in their brains. Labels of statistical comparisons (dependent samples *t*-tests): * = significant difference from pre-stimulus baseline (*p* < 0.05), ** = significant difference from pre-stimulus baseline (*p* < 0.01), *** = significant difference from pre-stimulus baseline (*p* < 0.001). For more details on the applied statistical approaches, please refer to the Methods section.

In Figure 2 we demonstrate the categorized electrophysiological effects on spontaneous firing rates in response to chemogenetic receptor activation, grouping them into distinct response modalities. Without grouping, the averaged neuronal response of all neurons in hM3D(Gq) rats yielded a highly significant neuronal inhibition that lasted at least 30 min post-injection (Figure 2C). Accordingly, the largest neuronal population in hM3D(Gq)-transduced animals in terms of response modality was the “inhibition”-type neurons, accounting for 28% of all recorded neurons (Figure 2A). These neurons exhibited significant reductions in firing rates upon DCZ administration (Figure 2E). However, we observed 4 additional larger neuronal groups (with ≥9% prevalence) that showed different responses to DCZ stimulation: excitation-inhibition (16%, Figure 2G), inhibition-excitation (11%, Figure 2I), excitation-inhibition-excitation (10%, Figure 2J) and excitation (9%, Figure 2K) type neurons.

#### hM4D(Gi) effects

We recorded and analyzed ACC neurons (*n* = 167) from hM4D(Gi)-transduced animals (*n* = 3) in response to DCZ administration. Neuronal excitation was the most frequent response observed (Figures 1B,E). This characteristic long-term excitatory response is demonstrated in Figure 1E, in a raw data sample, where all 3 recorded neurons radically increased their firing rate within a few minutes after the DCZ injection. On the raster diagram (Figure 1B), where the raw firing rates of all recorded neurons from hM4D(Gi)-transfected rats can be observed, an overwhelming excitatory response is evident, starting a few minutes after the DCZ injection and lasting until the end of the 55-min recording session.

When examining the typical spiking and oscillatory effects of DCZ in hM4D(Gi) -transduced animals (Figures 1F,H), we can observe the familiar ‘up-down’ cortical pattern discussed earlier. It’s important to note that in the case of hM4D(Gi) data, the y-axis scales in Figures 1F,H are compressed compared to Figures 1D,G, which allows us to visualize the opposite directional effects in both groups. In response to DCZ administration, we observed opposite effects to those seen in hM3D(Gq)-transduced animals. The “up-down” dynamics remained, the oscillatory power increased, and the neurons continued firing within the boundaries of the “up” states (Figures 1D,G, right panels). The largest neuronal population in hM4D(Gi)-transfected rats, in terms of response modality, was the “excitation”-type neurons, accounting for 53% of all recorded neurons (Figure 2B). These neurons exhibited a significantly increased firing rate in response to the DCZ injection (Figure 2F). Furthermore, this excitatory response remained significant even when considering the sum of neuronal responses from all recorded neurons (Figure 2D). There were no other considerably large neuronal classes; the next largest population consisted of ‘inhibition’ type neurons, representing only 6% of the entire neuronal population (Figure 2H).

#### Control recordings

Among the 130 neurons recorded from sham-operated control animals (*n* = 3) without any chemogenetic modifications applied in the brain, the majority exhibited no response to DCZ administration (Figure 2L). Specifically, 77% of these neurons fell into the ‘no effect’ category. However, there was a transient yet significant increase in the average firing rate immediately after DCZ injection during the initial 4 min when considering all neuronal responses. Additionally, a subtle shift towards higher firing activity was observed in the latter half of the recording session.

Moreover, it’s worth noting that the baseline firing rates in the −4-1 min before stimulation were lower in both viral-infected animal groups compared to the baseline frequency of control neurons (F_2,391_ = 30.954, *p* = 3.317 × 10^−13^; post-hoc Tukey HSD test: hM3D(Gq) vs. Sham *p* = 0.00002, hM4D(Gi) vs. Sham *p* = 0.00002).

### Effects on oscillatory LFP power

#### Effects on baseline-normalized oscillatory LFP power

Factorial ANOVA revealed that all three investigated factors were highly significant (DREADD: F_2,1980_ = 943.795, *p* < 10^−17^; LFP: F_4,1980_ = 75.804, *p* < 10^−17^; TIME: F_2,1980_ = 38.908, *p* < 10^−17^), as well as all interactions, including the triple interaction (*p* = 1.8 × 10^−10^). This was also the case when we analyzed the differences in a time-independent manner, with only two remaining variables (DREADD and LFP, all ANOVA’s *p* < 10^−17^).

**Figure 3:**
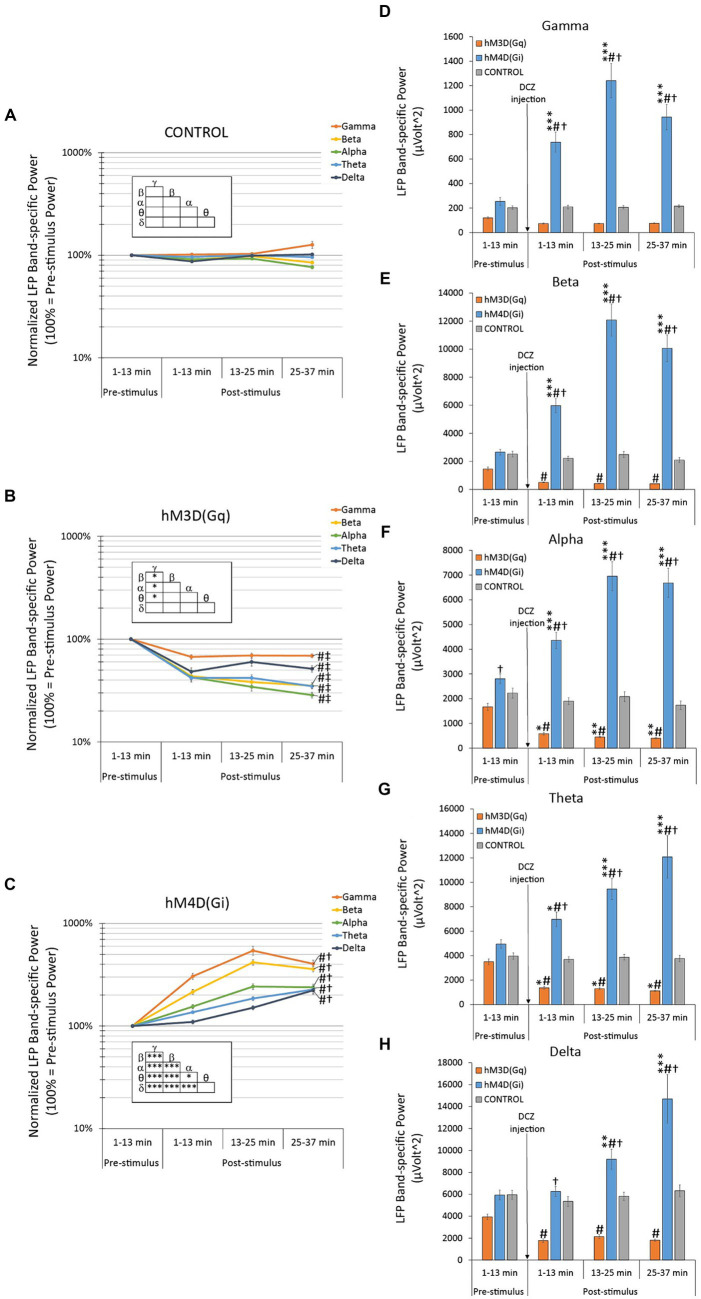
Effects of hM3D(Gq) and hM4D(Gi) DREADD receptor stimulation on baseline-normalized **(A–C)** and raw **(D–F)** oscillatory LFP power. **(A)** In control animals, with no available chemogenetic receptors in their brains, there was no statistically significant spontaneous change in the baseline-normalized oscillatory LFPs, nor any effect of the DCZ stimulation in any LFP bands during the entire recording session. **(B)** In hM3D(Gq) animals, there was a significant decrease in all baseline-normalized LFP bands, in response to DCZ stimulation. **(C)** In hM4D(Gi) animals, there was a significant increase in all baseline-normalized LFP bands, in response to DCZ stimulation. **(D–F)** Effects of DCZ stimulation on band-specific oscillatory LFP powers (**D**: gamma, **E**: beta, **F**: alpha, **G**: theta, **H**: delta). Labels of statistical comparisons: normalized data **(A–C)**: # = significant difference from the control group (*p* < 0.05), † = significant difference from the hM3D(Gq) group (*p* < 0.05), ‡ = significant difference from the hM4D(Gi) group (*p* < 0.05); within group differences (inserts): * = significant within-group difference (*p* < 0.05), ** = significant within-group difference (*p* < 0.01), *** = significant within-group difference (*p* < 0.001). Raw data **(D–F)** * = significant difference from pre-stimulus baseline (*p* < 0.05), ** = significant difference from pre-stimulus baseline (*p* < 0.01), *** = significant difference from pre-stimulus baseline (*p* < 0.001). # = significant difference from the control group within the same time interval (*p* < 0.05), † = significant difference from the hM3D(Gq) group within the same time interval (*p* < 0.05). For more details on the applied statistical approaches, please refer to the methods section.

In sham-operated control rats, there was no change in the baseline-normalized oscillatory LFP power throughout the entire recording session, nor any significant effect of the DCZ injection in any of the individual bands (Figure 3A). Consequently, no significant difference was observed between the bands either (Figure 3A insert).

In contrast, hM3D(Gq) animals showed a robust reduction (31–71% less than pre-DCZ levels) in the baseline-normalized oscillatory LFP power across all frequency bands (Figure 3B). These reductions were highly significant when compared to control animals with LSD post-hoc tests, indicating high statistical significance for the gamma and delta bands at *p* = 0.001 and *p* = 0.0008, respectively, and even higher significance for the beta, alpha, and theta bands (beta *p* = 0.00004, alpha *p* = 0.00005, and theta *p* = 6.0 × 10^−6^). Furthermore, when comparing hM3D(Gq) animals to the hM4D(Gi) group, LSD post-hoc tests similarly revealed highly significant differences across all frequency bands (all *p* < 10^−17^).

Within the hM3D(Gq) group, there were slightly significant variations. The reduction in baseline-normalized oscillatory LFP power was significantly less pronounced in the gamma-band when compared to all other bands (LSD post-hoc tests gamma vs. beta *p* = 0.028, vs. alpha *p* = 0.013, vs. theta *p* = 0.031), except the delta-band, which exhibited a similar, relatively smaller effect when subjected to DCZ administration (Figure 3B insert).

Remarkably, the oscillatory LFP power recorded from hM4D(Gi) animals exhibited a contrasting response when compared to hM3D(Gq) animals. A significant and pronounced increase in normalized oscillatory power was observed across all frequency bands (Figure 3C), reaching more than 500% values above baseline in the gamma band frequency range (i.e., 544% increase at post-DCZ 13–25 min). These increases were highly significant when compared to the control group, with LSD post-hoc tests confirming high statistical significance for all frequency bands (gamma, beta, alpha *p* < 10^−17^, theta *p* = 1.6 × 10^−12^, delta *p* = 7.0 × 10^−8^).

In contrast to the hM3D(Gq) group, the within-group analysis revealed substantial differences in the magnitude of response across all the frequency bands (Figure 3C insert). LSD post-hoc tests indicated significant variations between the highest affected gamma vs. the beta (*p* = 6.6 × 10^−13^), alpha (*p* < 10^−17^), theta (*p* < 10^−17^), and delta (*p* < 10^−17^) bands. Moreover, comparisons between the highly altered beta vs. the alpha (*p* < 10^−17^), theta (*p* < 10^−17^) and delta (*p* < 10^−17^) bands also demonstrated significant differences. The alpha band exhibited a slightly less significant change (*p* = 0.016) when compared to theta, but still was highly significant (*p* = 0.00002) in comparison with delta. Notably, the delta and theta bands displayed the least modulation in response to DCZ administration, and therefore, were statistically indistinguishable from each other.

#### Effects on raw oscillatory LFP power

##### All frequency bands

A factorial ANOVA analysis revealed highly significant effects for all three investigated factors (DREADD: F_2,2,640_ = 443.062, *p* < 10^−17^; LFP: F_4,2,640_ = 171.731, *p* < 10^−17^; TIME: F_3,2,640_ = 25.062, *p* = 5.5 × 10^−16^) and their interactions (*p* < 10^−5^ for all). These significant findings persisted when analyzing the individual frequency bands, the *p*-values for the “DREADD” (all *p* < 10^−17^) and “TIME” (all *p* < 0.001) factors, as well as their interaction (“DREADD × TIME”, *p* < 10^−17^), were highly significant. The exception was only the “TIME” factor in the theta band analysis, where it achieved a slightly lower, yet still highly significant *p*-value (*p* = 0.009).

Band-specific raw oscillatory LFP power responses to DCZ injection in hM4D(Gi) animals were significantly different from those in the hM3D(Gq) group across all frequency bands, as confirmed by LSD post-hoc tests (*p* < 0.001 in all cases; Figures 3D–H). Specifically, the oscillatory power within each frequency band consistently increased with time in hM4D(Gi)-transfected animals, while it consistently decreased in hM3D(Gq)-transduced rats.

Another noteworthy observation across all frequency bands in the raw data analysis is that the band-specific raw oscillatory power in the hM4D(Gi) group was consistently and significantly elevated compared to both the control group’s oscillatory power within the same band and the pre-DCZ oscillatory power (Figures 3D–H). In contrast, the changes in oscillatory power in the hM3D(Gq) group were less significant.

##### Gamma band

Remarkably, among all the frequency bands, the gamma band exhibited the most diverse response to DCZ administration between the hM4D(Gi) and hM3D(Gq) groups (Figure 3D). Specifically, while the gamma power displayed the largest and most significant increases among all frequency bands in hM4D(Gi) animals (LSD post-hoc tests *p* < 10^−7^) at all three timepoints vs. pre-DCZ levels, in hM3D(Gq) animals, the decrease in gamma power was the least pronounced among the frequency bands, not reaching statistical significance in the raw data analysis (Figure 3D) or normalized analysis, as elaborated on above (Figure 3B).

##### Beta band

In the hM4D(Gi) group, the beta band analysis yielded results similar to those seen in the gamma band, with a pronounced and highly significant increase in beta power following DCZ administration (Figure 3E, *p* < 10^−5^ at all three timepoints vs. pre-DCZ levels).

##### Alpha band

In the hM3D(Gq) group, responses to DCZ activation in the alpha band raw data exhibited the most significant reduction in power among all the frequency bands (Figure 3F). This reduction was not only significant when compared to the control group, as demonstrated by LSD post-hoc tests (1–13 min post-DCZ, *p* = 0.003; 13–25 min post-DCZ, *p* = 0.0003; 25–37 min post-DCZ, *p* = 0.003), but it was also significant when compared to the pre-DCZ injection values (1–13 min post-DCZ, *p* = 0.021; 13–25 min post-DCZ, *p* = 0.010; 25–37 min post-DCZ, *p* = 0.007).

##### Theta band

DCZ’s effects on raw theta were comparable to those on alpha, with one notable difference being that effects on both hM3D(Gq) and hM4D(Gi) exhibited slightly reduced magnitudes (Figure 3G).

##### Delta band

When considering all factors, the delta band power was the least affected by DCZ injection (Figure 3H). While still reflecting the across-band characteristic responses, namely the significant increase in oscillatory power in hM4D(Gi) and the significant decrease in hM3D(Gq), the significance of the former effect was lost compared to both the control group and the pre-DCZ baseline in the first time interval (1–13 min post-DCZ). Similarly, in hM3D(Gq) rats, the significance compared to pre-injection oscillatory power values disappeared, while the significant difference compared to control values remained (LSD post-hoc tests vs. controls: 1–13 min post-DCZ, *p* = 0.003; 13–25 min post-DCZ, *p* = 0.002; 25–37 min post-DCZ, *p* = 0.0002).

### Effects on spiking-associated oscillatory LFP power

In a separate analysis, we modified the approach used for oscillatory LFP power analysis, as previously discussed and illustrated in Figure 3. However, in this case, we narrowed our focus to the ±200 ms peri-injection LFP signal in close proximity to individual neuronal spikes (action potentials). The purpose of this tailored analysis is to specifically examine changes in oscillatory LFP power that are synchronized with the spontaneous firing activity of local neurons. This method provides an emphasized view on the network oscillations that are locally affected by chemogenetic manipulation.

#### Effects on baseline-normalized spiking-associated oscillatory LFP power

A factorial ANOVA revealed the high significance of all three examined factors: DREADD (F_2,4,920_ = 1345.328, *p* < 10^−17^), LFP (F_4,4,920_ = 80.746, *p* < 10^−17^), and TIME (F_2,4,920_ = 45.778, *p* < 10^−17^). Moreover, all interactions between these factors, including the triple interaction, were found to be highly significant (*p* < 0.00003 for all). This significance persisted even when we conducted the analysis in a time-independent manner, considering only two remaining variables (DREADD and LFP), where all ANOVAs remained highly significant (*p* < 10^−17^).

**Figure 4:**
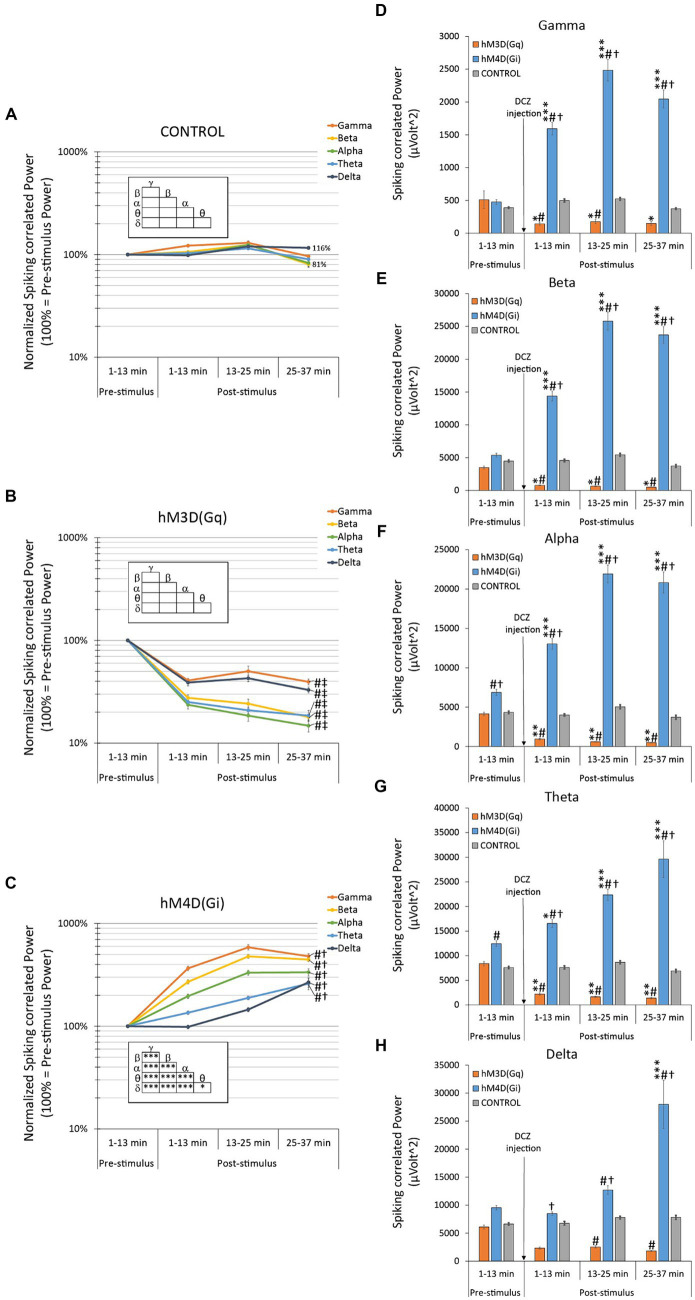
Effects of hM3D(Gq) and hM4D(Gi) DREADD receptor stimulation on baseline-normalized **(A–C)** and raw **(D–F)** spiking-associated oscillatory LFP power. This analysis is similar to the one in Figure 3, except that only the ±200 ms peristimulus LFP signal was analyzed related of each single neuronal spikes (action potentials). **(A)** In control animals, with no available chemogenetic receptors in their brains, there was no statistically significant spontaneous change in the baseline-normalized spiking-associated oscillatory LFPs, nor any effect of the DCZ stimulation in any LFP bands during the entire recording session. **(B)** In hM3D(Gq) animals, there was a significant decrease in all baseline-normalized spiking-associated LFP bands, in response to DCZ stimulation. **(C)** In hM4D(Gi) animals, there was a significant increase in all baseline-normalized spiking-associated LFP bands, in response to DCZ stimulation. **(D–F)** Effects of DCZ stimulation on band-specific oscillatory LFP powers (**D**: gamma, **E**: beta, **F**: alpha, **G**: theta, **H**: delta). Labels of statistical comparisons: normalized data (**A–C**): # = significant difference from the control group (*p* < 0.05), † = significant difference from the hM3D(Gq) group (*p* < 0.05), ‡ = significant difference from the hM4D(Gi) group (*p* < 0.05); within group differences (inserts): * = significant within-group difference (*p* < 0.05), ** = significant within-group difference (*p* < 0.01), *** = significant within-group difference (*p* < 0.001). Raw data **(D–F)** * = significant difference from pre-stimulus baseline (*p* < 0.05), ** = significant difference from pre-stimulus baseline (*p* < 0.01), *** = significant difference from pre-stimulus baseline (*p* < 0.001). # = significant difference from the control group within the same time interval (*p* < 0.05), † = significant difference from the hM3D(Gq) group within the same time interval (*p* < 0.05). For more details on the applied statistical approaches, please refer to the Methods section.

Animals expressing hM3D(Gq) exhibited a drastic decrease (60–85% lower than pre-DCZ levels) in spiking associated baseline-normalized oscillatory LFP power across all frequency bands (Figure 4B vs. there were no changes in control animals depicted in Figure 4A). Notably, these reductions were more pronounced than those observed in the overall analysis presented in Figure 3B, and, in contrast to the original analyses, there were no significant differences between the frequency bands within the hM3D(Gq) group (Figure 4B insert).

The spiking-associated oscillatory LFP power analysis in hM4D(Gi) animals also demonstrated an inverse response compared to that seen in hM3D(Gq) animals. A significant and radical increase in normalized oscillatory power was observed across all frequency bands (Figure 4C), reaching values exceeding 500%, particularly evident in the gamma band frequency range (i.e., a 587% increase at 13–25 min post-injection).

#### Effects on raw spiking-associated oscillatory LFP power

Since the outcome of the analysis on this subset of data was very similar to the observations we made above, in the full data set analysis (Figures 3D–H and 4.2.2. “Effects on raw oscillatory LFP power”), we are only going to highlight the substantial differences between the results coming from the two different approaches.

Consistent with the full data set analysis (Figures 3D–H), we observed that the band-specific spiking-associated raw oscillatory power in the hM4D(Gi) group was persistently and significantly elevated compared to both the control group’s oscillatory power and the pre-DCZ oscillatory power (Figures 4D–H). However, in contrast to the original analysis, the opposing directional changes in the hM3D(Gq) group became more pronounced, reaching statistical significance compared to the pre-DCZ baseline not only in the alpha and theta frequency bands, but additionally in the gamma and beta bands (Figures 4D,E). Furthermore, both analyses converged on the conclusion that delta band power exhibited the least sensitivity to DCZ administration.

### Possible confounding effects of sedation

To demonstrate that low-dose isoflurane sedation did not significantly confound our results, we conducted pilot experiments by replacing isoflurane with ketamine as the primary anesthetic. Ketamine operates through a distinct mechanism of action, interacting with N-methyl-D-aspartate (NMDA), opioid, monoaminergic, and muscarinic receptors, in contrast to isoflurane, which primarily engages with GABA receptors (Chung et al., 2013). Under ketamine anesthesia, we observed the typical increase in firing rates, accompanied by the characteristic enhancement of oscillatory LFP activity in response to hM4D(Gi) stimulation (Supplementary Figure S3). This pattern closely mirrored what was observed under isoflurane sedation.

### Histology

**Figure 5:**
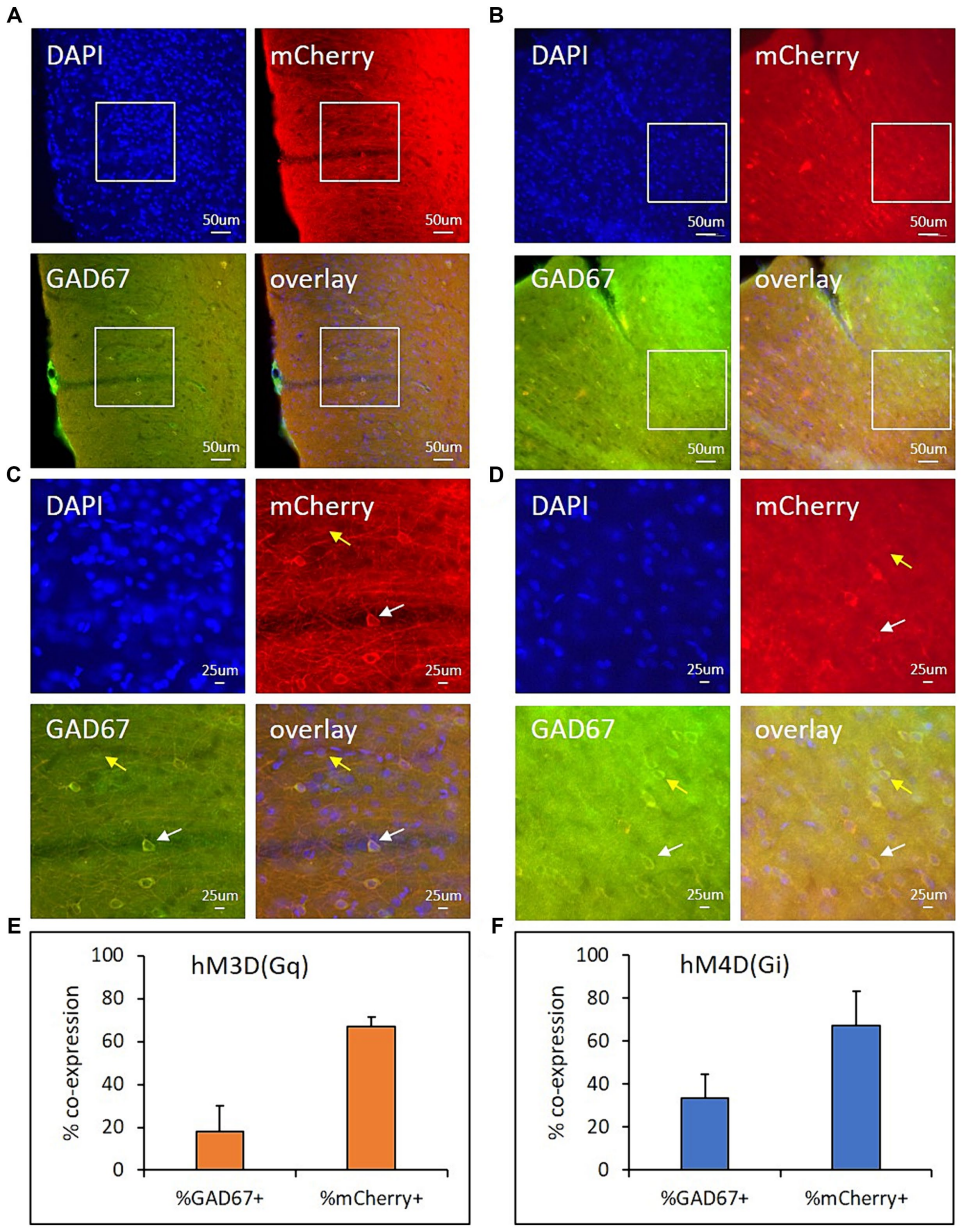
hM3D(Gq) and hM4D(Gi) expression in GABAergic cells within the ACC. Representative images from rats expressing hM3D(Gq) **(A,C)** or hM4D(Gi) **(B,D)** at 20x **(A,B)** magnification in the ACC. **C,D** are zoomed in representative regions of quantification (200 μm × 200 μm, indicated by white boxes in **A,B**). DAPI (first panels, blue) fluorescence represents cell nuclei. mCherry (second panels, red) fluorescence represents viral transduction. GAD67 (third panels, green) fluorescence represents GABAergic cell expression. The last panels for **A–D** (overlay of all 3 channels) illustrates viral vector transduction (mCherry) within GABAergic (GAD67+) and non-GABAergic (GAD67-) cells in the ACC. Bar graphs show the average %GAD67+ cells that co-express mCherry (**E,F**: first bars) and the average %mCherry+ cells that co-express GAD67 (**E,F**: last bars) hM3D(Gq) (orange) and hM4Di(Gi) (blue) animals (3 sections/rat). Scale = 50 um **(A,B)** or 25 um **(C,D)**. White arrows **(C,D)** indicate mCherry+/GAD67+ cells. Yellow arrows **(C,D)** indicate mCherry-/GAD67+ cells.

Viral injections into the ACC region were confirmed through the visualization and mapping of viral vector-tagged mCherry fluorescence. Representative images from an hM3D(Gq)- (Figures 5A,C) and an hM4D(Gi)- (Figures 5B,D) transduced animal are shown in Figure 5, demonstrating expression of cell nuclei (DAPI), viral transduction (mCherry), and GABAergic cells (GAD67) in the ACC. Quantification of a subregion within the transduced area of the ACC (indicated by white box in Figures 5A,B and shown in Figures 5C,D) revealed that 17.98 ± 12.09% of all GAD67+ cells were transduced with hM3D(Gq) and 33.52 ± 10.82% of all GAD67+ cells were transduced with hM4D(Gi) (Figures 5E,F). Additionally, 67.04 ± 4.42% hM3D(Gq)-transduced cells co-expressed GAD67 and 67.15 ± 16.02% hM4D(Gi)-transduced cells co-expressed GAD67 (Figures 5E,F). Overall, these results indicate that the majority (~67%) of transduced cells within the ACC are inhibitory, GABAergic cells.

## Discussion

This study investigated the *in vivo* electrophysiological effects of commonly used DREADDs, hM4D(Gi) and hM3D(Gq), on cortical spontaneous firing rate and LFP oscillations in rats under light anesthesia. The primary aim was to confirm that hM4D(Gi) predominantly silences neurons while hM3D(Gq) mainly activates neurons in the cortex. However, unexpected inhibitory effects were observed with pan-neuronal hM3D(Gq) stimulation, and excitatory effects with pan-neuronal hM4D(Gi) stimulation using the actuator DCZ.

Pan-neuronal hM3D(Gq) activation resulted in predominantly inhibitory effects *in vivo*, contradicting the anticipated increased excitability reported in cellular/molecular-level studies (Armbruster et al., 2007; Krashes et al., 2011). Similarly, the effects of chemogenetic manipulation on spontaneous firing activity in hM4D(Gi)-transduced animals were unexpected. The largest neuronal population in this group exhibited excitation-type responses, accounting for 53% of all recorded neurons, while inhibitory modality neurons represented only 6% of the total recorded neuronal population. Similar counterintuitive outcomes in DREADD effects have been documented in various earlier studies (Vazey and Aston-Jones, 2014; Chang et al., 2015; Deffains et al., 2021; Rogers et al., 2021). These prior findings together with our current results challenge the notion that pan-neuronal hM3D(Gq) DREADDs serve as universal excitatory agents on the neuronal network level, or that targeted hM4D(Gi) DREADDs could effectively ‘switch off’ complete brain regions.

Previous studies have not explored the impact of hM3D(Gq) and hM4D(Gi) activation on cortical spiking-oscillation coupling and ‘up-down’ oscillatory dynamics. These cortical patterns have been observed in rodents and monkeys not only during sleep but in wakefulness and showed that ‘up-down’ dynamics play a crucial role in regulating cognitive tasks and quiescent behavior (Jercog et al., 2017). We demonstrate that pan-neuronal hM3D(Gq) activation leads to a significant decrease in ‘up-down’ rhythmicity and oscillatory power, likely due to neurons firing outside of ‘up’ states, quickly after the DCZ stimulation (<1 min). This phenomenon may result from increased excitability of some neurons in the network, following hM3D(Gq) activation, and could contribute not only to the decreased up-down rhythmicity but also partially explain the observed reduction in oscillatory power (via decreased synchronicity). On the contrary, hM4D(Gi) activation resulted in contrasting effects on spiking-oscillation coupling and up-down oscillatory dynamics. Specifically, following DCZ administration, the ‘up-down’ dynamics persisted, accompanied by an increase in oscillatory power, and neurons continued firing within the ‘up’ states. These results highlight that pan-neuronal chemogenetics can influence not only spiking frequencies but also affect broader oscillatory dynamics.

Our findings also demonstrate that cortical pan-neuronal chemogenetic manipulations can significantly alter local oscillatory LFP powers, impacting all frequency bands from delta to gamma. In hM4D(Gi) animals, there were robust increases in normalized oscillatory power across all frequency bands, with values exceeding 5 times baseline levels in the gamma band after DCZ administration. Conversely, in the hM3D(Gq) group, responses were reversed compared to hM4D(Gi) animals, with oscillatory power consistently decreasing across all bands following DCZ administration. Upon further examination of the chemogenetic effects on oscillatory LFP power, our study reveals differences among frequency bands. For example, the gamma band exhibited the most varied responses, with hM4D(Gi) animals showing the largest and most significant increases compared to other frequency bands, while hM3D(Gq) animals displayed the least pronounced effects on gamma power, whereas delta band power appeared to be the least affected in both groups.

Spiking-oscillation coupling, especially neuronal spiking synchronized to theta activity, is proposed to facilitate coherent communication between different cortical areas, such as the ACC and the hippocampus (Buzsáki, 2002; Womelsdorf et al., 2010). Theta oscillations, crucial for working memory, can modulate local high-frequency gamma band synchronization nested within the theta cycle. Impaired theta-gamma cross-frequency coupling is implicated in various cognitive processes, including attention, learning, and memory, as well as disorders like epilepsy and Alzheimer’s Disease (Goodman et al., 2018; Kitchigina, 2018; Park et al., 2020), highlighting its pathophysiological relevance in humans. Theta synchronization is also implicated in playing a role in several learning-related network processes (Likhtik et al., 2014; Chen et al., 2021). Therefore, based on our current results demonstrating the capability of hM3D(Gq) and hM4D(Gi) activation to drastically modify spiking-oscillation coupling, ‘up-down’ oscillatory dynamics, and power of cortical theta and gamma oscillations, we hypothesize that the chemogenetic approach, in general, could be effectively applied to influence various neurophysiological processes where the alteration of local network oscillations or their coupling with spiking activity could be a crucial research or therapeutic goal. We hypothesize that beyond single-neuron excitability modification, chemogenetic methods have the potential to modify the complex functionality of local cortical networks, including the targeted neuromodulation of oscillatory functions. In this way, pan-neuronal or targeted chemogenetic manipulations might be investigated in the future as possible new therapeutic approaches, effectively improving several neurological disorders, such as epilepsy, Alzheimer’s Disease or other cognitive impairments.

**Figure 6:**
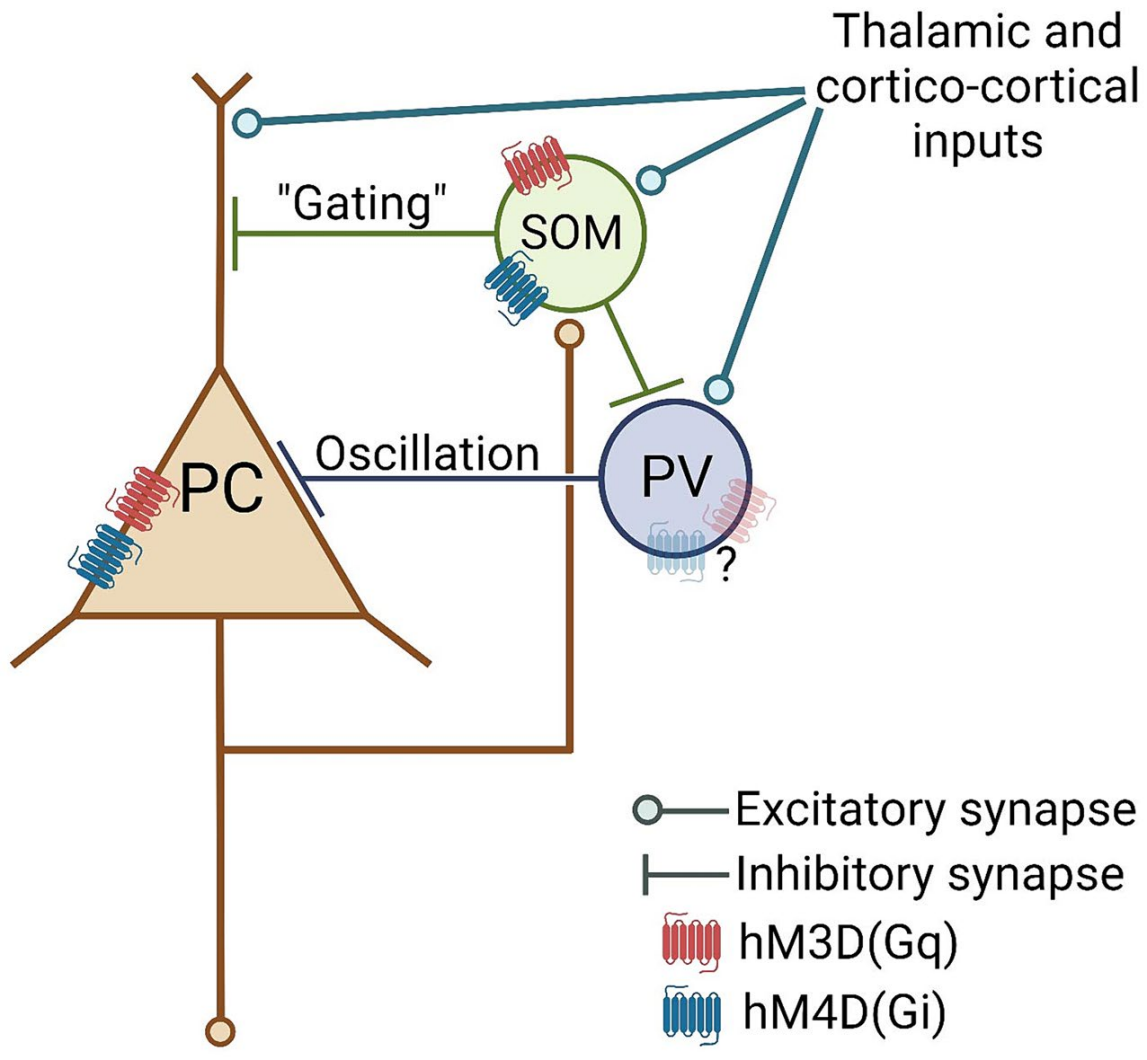
Hypothetical cortical network model with plausible sites of perturbatory action of pan-neuronal hM3D(Gq) and hM4D(Gi) DREADD receptor stimulation. PC, Principal Cell; PV, parvalbumin interneuron; SOM, somatostatin interneuron.

Some potential mechanisms underlying the hereby demonstrated perturbation effects resulting from chemogenetics have previously been proposed in the literature (Smith et al., 2016). For instance, considering that not all neurons express the chemogenetic receptors in the targeted brain area, non-expressing cells might still exhibit indirect electrophysiological changes in their firing activity, even if not directly influenced by the actuator molecule (e.g., DCZ in our case). Acknowledging the significance of parvalbumin (PV)- and somatostatin (SOM)-positive GABAergic interneurons as crucial components of cortical networks, including the ACC (Liguz-Lecznar et al., 2016; Tremblay et al., 2016; Guet-McCreight et al., 2020; Shao et al., 2021; Keijser and Sprekeler, 2023), it is plausible that local GABAergic neurons have the capacity to modify or even reverse the effects of excitatory or inhibitory chemogenetic manipulation, which would be an unintended consequence of experimenter use of chemogenetics. Consequently, in cortical brain areas, it is likely that at least a portion of the anticipated chemogenetic neuromodulatory effects will be reversed by local intra-area GABAergic circuits. As a theoretical example, one potential network mechanism explaining our current results is depicted in Figure 6 (for a more detailed model, refer to Supplementary Figure S4). Based on our findings that the majority of the virally-transduced cells in the ACC are GABAergic, it is likely that, along with principal neurons, cortical inhibitory interneurons do express a sufficiently large number of functional DREADDs, which, in turn, can “switch” the molecular action of hM3D(Gq) and hM4D(Gi) receptors in the neocortex on the network level.

For additional support of this “interneuronal switch” hypothesis, it’s worth noting that due to the technical nature of our recording electrodes (further details can be found in the “Limitations” section), our recorded dataset appears to be somewhat biased towards pyramidal cells. Consequently, the counterintuitive electrophysiological effects observed, primarily affecting principal neurons, may potentially stem from indirect interneuronal DREADD stimulations. Moreover, the plausible underlying “direct” interneuronal effects, such as hM3D(Gq)-mediated excitation and hM4D(Gi)-mediated inhibition, might have remained somewhat obscured by our recording electrode.

Our secondary objective was to explore the short-term *in vivo* electrophysiological pharmacodynamics of systemically administered DCZ, to further prove its feasibility in *in vivo* applications. According to our knowledge, this study provides the first demonstration of the *in vivo* electrophysiological effectiveness of DCZ, as a new and promising chemogenetic actuator drug. As CNO has been demonstrated to be pharmacologically sub-optimal for the precise activation of chemogenetic receptors in the brain, in accordance with previous reports (Jendryka et al., 2019; Nagai et al., 2020; Nentwig et al., 2022), this investigation provides further supporting evidence for the use of DCZ in chemogenetic experiments. In line with previous rat data (Nentwig et al., 2022), we have found that the chosen 0.3 mg/kg i.p. administered DCZ dose was a reasonably low and reliably effective dose in rats. DCZ seems to be effectively penetrating the brain and causing fast electrophysiological action on cortical neurons, within a few minutes after systemic application.

### Limitations

The use of anesthetics in our study has the potential to introduce unknown and uncontrolled confounding effects. However, the fact that we replicated our results using a different anesthetic with a different neuronal mechanism of action (Supplementary Figure S3) somewhat rules out the pivotal role of the anesthetic mechanism significantly affecting our observations. Nevertheless, follow-up experiments on awake and freely behaving animals would be valuable to confirm our current observations.

Our study observed decreased baseline firing activity in viral-infected animals compared to controls without chemogenetic receptors. While virus titers were within safe limits (10^13^ vg/mL) (Wirtshafter and Stratford, 2016; Goossens et al., 2021), we cannot rule out potential neurotoxic effects. Limited data exist on the long-term consequences of chemogenetic applications, emphasizing the need for further research to optimize safety and efficacy.

Due to the technical nature of our recording setup, we were generally not able to record the firing activity of fast-spiking (>50 Hz) PV interneurons (Nahar et al., 2021). This lack of PV interneuronal data in our sample could be related to the employed extracellular multichannel recording probes (A1x16-5 mm-150-177, NeuroNexus Co., Ann Arbor, MI), which typically have somewhat low impedance (approx. 0.5–3 MOhm). This impedance range is suggested to be strongly biased toward recording form pyramidal cells (Moore and Wehr, 2014).

## Conclusion

In conclusion, our investigation of the *in vivo* electrophysiological effects of the commonly used DREADDs, hM4D(Gi) and hM3D(Gq), in slightly sedated rats yielded unexpected outcomes regarding spontaneous firing rates and novel findings relating to LFP oscillations. Contrary to predictions, with DCZ-activation, pan-neuronal hM3D(Gq) activation predominantly induced inhibitory effects, challenging the notion of its intended universal excitatory actions. Similarly, hM4D(Gi) activation led to unexpected excitatory responses in the majority of recorded neurons. The possible underlying reasons of these unexpected electrophysiological effects include the inefficient expression of chemogenetic receptors within the targeted cortical area, and the observed transduction preference towards GABAergic interneurons over principal neurons. This study emphasizes the need to account for perturbation effects on the network level, particularly when using pan-neuronal promoters in chemogenetic applications.

Moreover, our study reveals that pan-neuronal cortical chemogenetic modulation can profoundly alter oscillatory neuronal activity, presenting a potential research tool or therapeutic strategy in neuropsychiatric models and diseases where oscillatory functions play a role.

## Data availability statement

The raw data supporting the conclusions of this article will be made available by the authors, without undue reservation.

## Ethics statement

The animal study was approved by Pennsylvania State University Institutional Animal Care and Use Committee. The study was conducted in accordance with the local legislation and institutional requirements.

## Author contributions

PK: Conceptualization, Data curation, Formal analysis, Investigation, Methodology, Writing – original draft. LB: Conceptualization, Data curation, Formal analysis, Methodology, Writing – review & editing. NZ: Funding acquisition, Writing – review & editing.
