# Supplementary figures and images for: Perturbing cortical networks: in vivo electrophysiological consequences of pan-neuronal chemogenetic manipulations using deschloroclozapine
Source: Front Neurosci. 2024 Apr 25;18:1396978. doi: 10.3389/fnins.2024.1396978 (PMC11079238; doi:10.3389/fnins.2024.1396978)

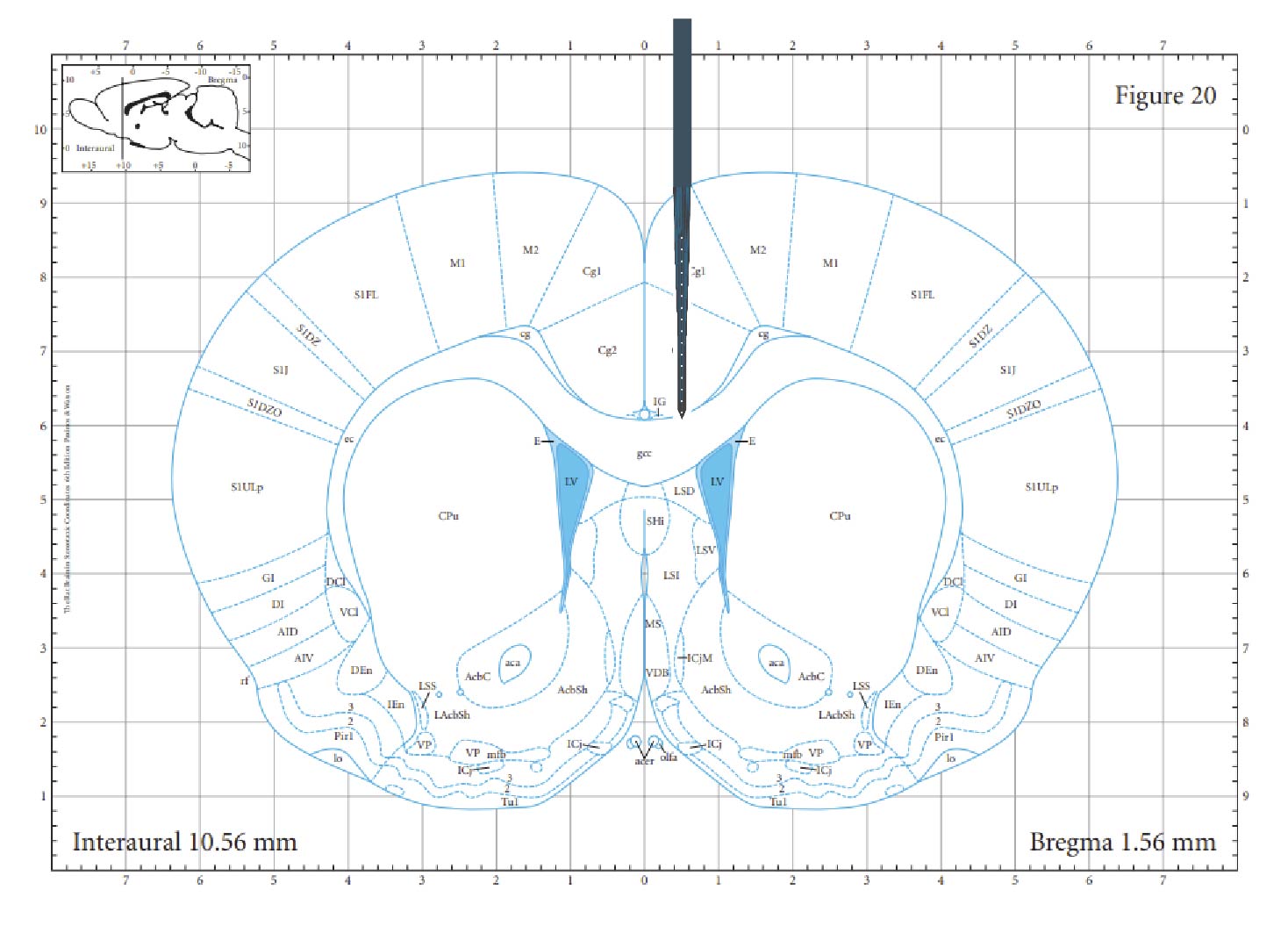

Supplement: Supplementary file 1 [file Image_1.JPEG]

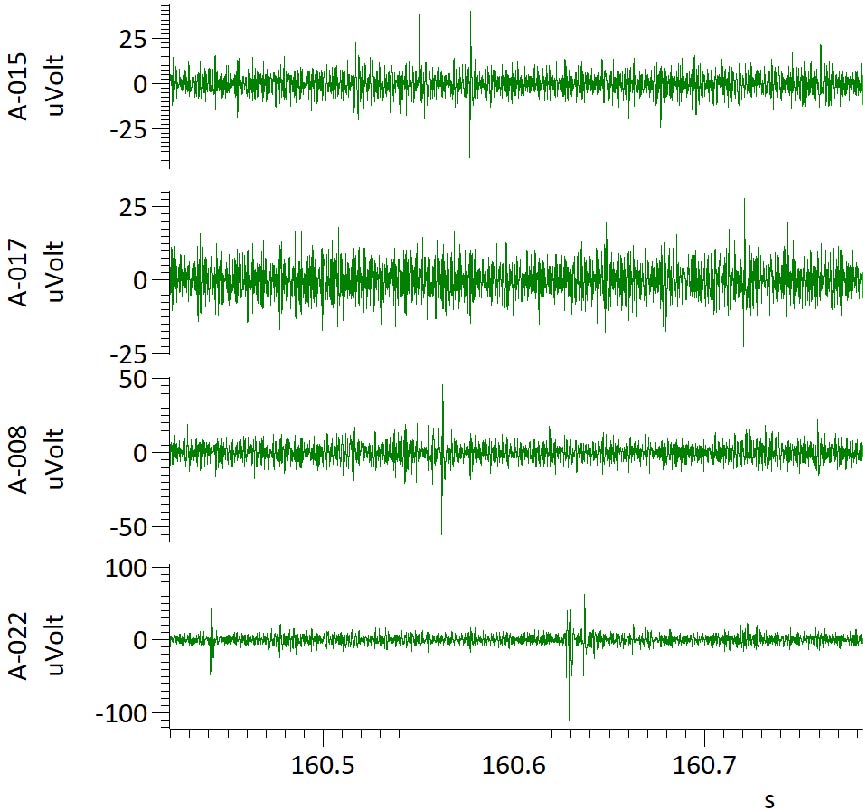

Supplement: Supplementary file 2 [file Image_2.JPEG]

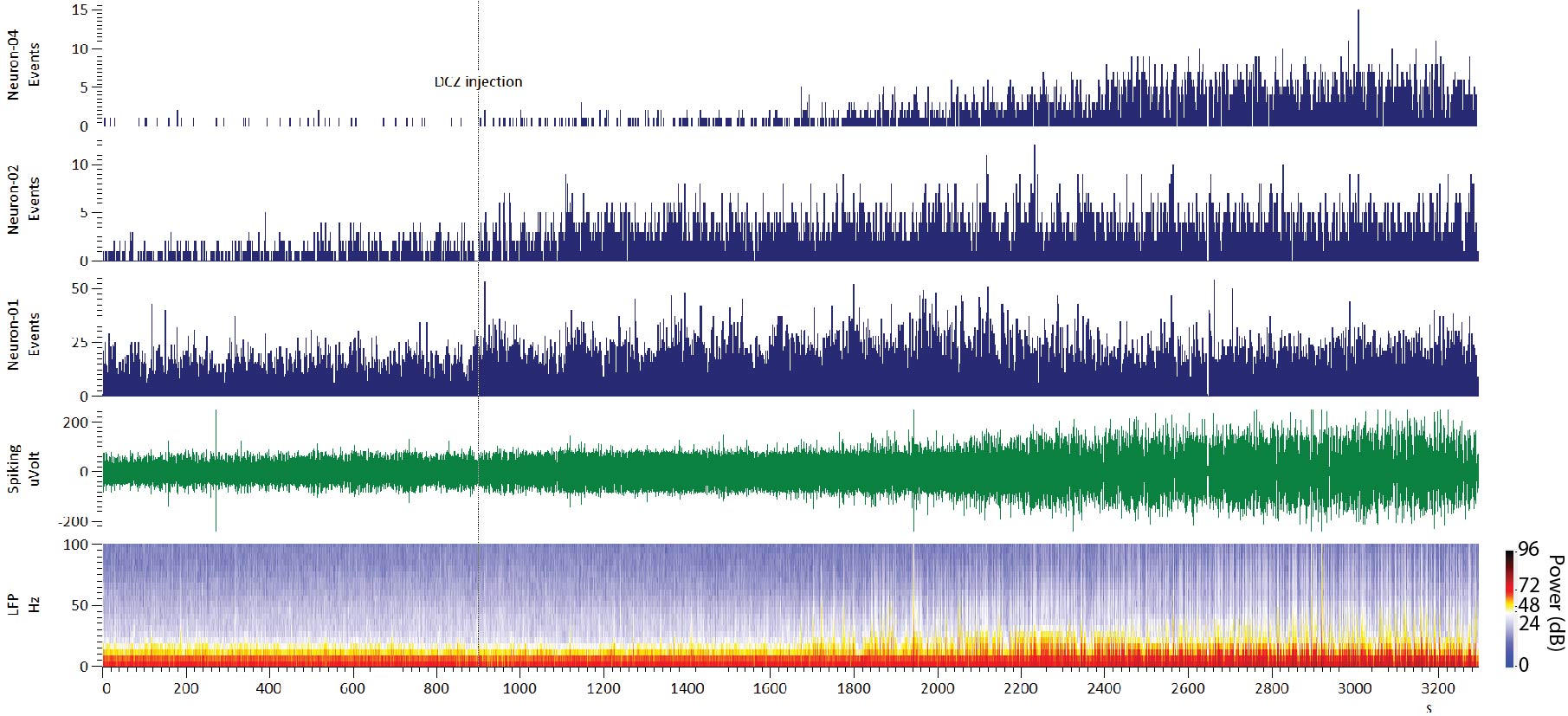

Supplement: Supplementary file 3 [file Image_3.JPEG]

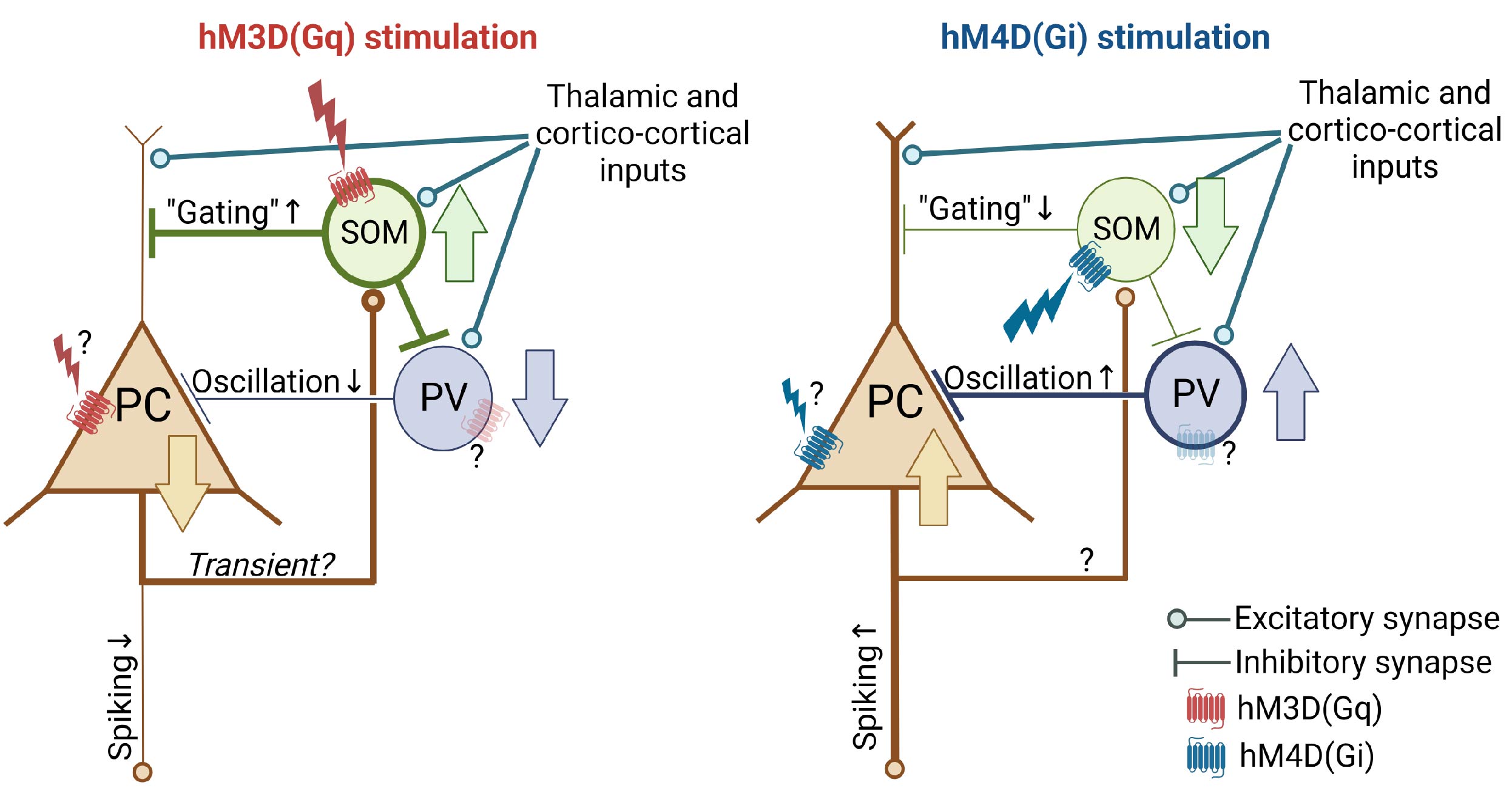

Supplement: Supplementary file 4 [file Image_4.JPEG]
